# Optimization of Biodesulfurization of sour heavy crude oil

**DOI:** 10.1371/journal.pone.0283285

**Published:** 2023-04-04

**Authors:** Wisam Mohammed Kareem Al-khazaali, Seyed Ahmad Ataei

**Affiliations:** Faculty of Engineering, Department of Chemical Engineering, Shahid Bahonar University of Kerman, Kerman, Iran; Universidad Tecnica de Manabi, ECUADOR

## Abstract

Biodesulfurization of fossil fuels is a promising method for treating the sour oil due to its environmental friendliness and ability to get rid of the recalcitrant organosulfur compounds. In this study, many types of microorganisms such as *Ralstonia* eutropha, *Rhodococcus* erythropolis, *Acidithiobacillus* ferrooxidans, *and Acidithiobacillus* thiooxidans applied on a sour heavy crude oil (sulfur content was 4.4%). Also, a colony isolated from the crude oil and oil concentrate was examined by supplying it with PTCC 106. The various official and famous mediums were significantly evaluated such as (PTCC 2, PTCC 105, PTCC 106 (9K), PTCC 116, PTCC 123, PTCC 132), sulfur-free MG-medium, basal salts medium, and mineral salts. It was found that *Rhodococcus erythropolis* and *Acidithiobacillus ferrooxidans* from microorganisms and SFM and the medium PTCC 105 were selected as the higher desulfurization efficiencies of crude oil equaling 47 and 19.74% respectively. The bioreactions depend on the treated fluid, targeting sulfur compounds as these represent the environmental status (amounts and types of nutrients), and the type of biotreaters whether microorganism are septic, semiseptic, or aseptic. The optimum operation conditions have been designed by using Definitive method such as mixing speed, temperature, surfactant dose, OWR, acidity. The optimum efficiencies obtained here are better than the previous efforts even though those gained by bioengineering. Biodesalination was a simultaneous process with the BDS.

## Introduction

Fossil Fuel is an important source of energy or power in various fields in life and industry. Before applying it in customization use, it must be on specification of some related standards to avoid risks on HSE. Then, sulfur compounds are one of these constraints to be treated, or else, they formulate a gangerence on quality and HSE limitations heart diseases, asthma, and respiratory illnesses [1].

There are many methods of treatment such as adsorptive desulfurization-adsorption (ADS) [2], microbial and bacterial desulfurization (BDS) [3], Extraction (Desulfurization by Extraction, EDS) [4], ODS-Oxidative Desulfurization (Desulfurization by oxidation DO) [1], supercritical water-based (water) desulfurization (SWD) [5]. Also, a combination is such as BDS-ODS-EDS [6], EDS-HDT [7], OEDS (Oxidation Extraction Desulfurization), microwave catalytic hydrogenation process [6].

BDS is more efficient and less expensive than the remaining methods as HDS in removing sulfur from refractory heterocyclic compounds present in crude oil, it could be used in oil refineries as a complement to achieve ULSD. Indeed, BDS can be used to desulfurize heavy oils, as shale oils, which have high thiophene concentration [8].

Few studies and patents were subjected on a real fractions as the whole crude oil in aerobic or anaerobic conditions by *Achromobacter*, *Leptospirillum*, *Pseudomonas*, *Sulfolobus*, *Thiobacillus*, *Rhodococcus* strains, *Sphingomonas* subarctica, *Bacillus*, *Desulfovibrio* desulfuricans, *Pyrococcus*, *Desulfomicrobium* scambium, *Desulfovibrio* longreachii, *Pantoea* agglomerans [9,10]. The advantage of BDS of whole crude oil is to reduce the costs of desulfurization treatment in refineries [11]. Also, BDS could be applied on the oil derivatives such as the LPG, petrol (gasoline), jet fuel (Aeroplane), kerosene, fuel oil (heavy oil of furnace or reboiler), and gas oil, and cracked stocks were applied to the BDS by these various microorganisms *Mycobacterium goodii*, *Pseudomonas*, *Gordonia*, *Rhodococcus*, *Mycobacterium phlei*, *Pseudomonas delafieldii*, *Paenibacillus*, *Rhodoccocus globerulus*, Nocardia [11,12].

Furthermore, a model compounds can be applied to lump and represent the whole fossil fuel especially the recalcitrant HCS [13] such as BT, DBT, DBTO2,M DBTSO2, MgSO4, BNT, DBS, 2,8 DMDBT, 2,6 DNDBT, DMDBT, thianthrene [8,14–23]. Finally, the microorganisms of BDS applied on water or coal could be common in application of oil [24].

## Materials and methods

### Materials

Nutrient components: There are many chemical components required in the preparation of culture mediums of microorganisms in order to achieve the growth and desulfurization. According to the inventory of recipe of nutrient media in appendix, the chemical species can be divided into carbohydrates, minerals, proteins and vitamins, furthermore, adaptation agents, acids and bases as HCl NaOH, and surfactants (Tween polysorbate 80 C_64_H_124_O_26_ and PEG as emulsification and demulsification reagents respectively [25].

Six official types of mediums were prepared by using the chemical species: medium PTCC 105, medium PTCC 106 (9K), medium PTCC 119, medium PTCC 123, medium PTCC 132, and medium PTCC 69 ‘Nutrient Broth (LB)’. Also, three typical mediums were applied: sulfur free mineral medium SFM [3], basal salt medium (BSM), mineral salt medium (MS). These mediums were prepared for seed culture of the microorganisms.

Then, the applied mediums were as follows (per 1000 ml of distilled water): PTCC **2** (pH 7) “LB, NB”: CaCl_2_ 5 g, peptone 5 g, meat extract 3 g, MnCl_2_.2H_2_O 10 mg, CaCl_2_.2H2O 100 mg, MgSO_4_.7H_2_O 500 mg, pH 7; PTCC 105 (pH 1.4) “ferrous sulfate medium *Theobacillus ferroxidans* medium with ferrus sulfat”: FeSO_4_ 33.33 g, KH_2_PO_4_ 0.4 g, MgSO_4_.7H_2_O 0.4 g, (NH_4_)_2_SO_4_ 0.4 g; PTCC 106 (pH 2) “9k medium”: FeSO_4_.7H_2_O 45 g, glucose 10 g, (NH_4_)_2_SO_4_ 3 g, K_2_HPO_4_ 0.75 g, MgSO_4_.7H_2_O 0.5, KCl 0.1 g, CaNO_3_ 0.01 g; PTCC 116 (pH 7) “Propionibacterium Medium”: elemental sulfur 10 g, KH_2_PO_4_ 3 g, CaCl_2_.2H_2_O 0.14 g, NH_4_Cl 0.1 g, MgCl_2_.6H_2_O 0.1 g; PTCC 123 (pH 3.5) “MS medium”: elemental sulfur 5 g, (NH_4_)_2_SO_4_ 2 g, K_2_HPO_4_ 0.25 g, MgSO_4_.7H_2_O 0.25 g, KCl 0.1 g; PTCC 132 (pH 1.8) “Leptospirillum (HH) Medium”: CaCl_2_.2H_2_O 147 mg, (NH_4_)_2_SO_4_ 132 mg, ZnCl_2_ 68 mg, CaCl_2_.6H_2_O 64 mg, MgCl_2_.2H_2_O 62 mg, MgCl_2_.6H_2_O 53 mg, KH_2_PO_4_ 27 mg, FeSO_4_ 20 mg.

Also, the following mediums were examined: MG-medium sulfur free mineral medium “SFM” (pH 7): glucose 5 g, NH_4_Cl 1 g, KH_2_PO_4_ 2 g, K_2_HPO_4_ 4 g, MgCl_2_.6H_2_O 0.2 g, TES 10 ml; whereas, TES: HCl (10 M) 10 ml, CaCl_2_.2H_2_O 2 g, NaCl 1 g, ZnCl_2_ 0.5 g, MnCl_2_.4H_2_O 0.5 g, CuCl_2_.2H_2_O 0.5 g, FeCl_2_.4H_2_O 0.4, Na_2_WO_4_.2H2O 0.1 g, Na_2_MoO_3_.2H_2_O 0.05 g [26]; BSM (PH 7) “BSM”: MnSO_4_.7H_2_O 2.2 g, KH_2_PO_4_ 1 g, K_2_HPO_4_ 1 g, NaNO_3_ 1 g, MgSO_4_.7H_2_O 0.5 g, CaCl_2_.9H_2_O 0.1 g. TES: ZnSO_4_.7H_2_O 2.2 g, MnSO_4_ 2 g, H_3_BO_3_ 0.4 g, CuSO_4_ 0.26 g, Na_2_MoO_4_.4H_2_O 0.26 g, CaCl_2_ 0.22 g, KI 0.006 g [27]; MS “mineral salts”: Na_2_HPO_4_.12H_2_O 3.57 g, KH_2_PO_4_ 1.5 g, H_3_BO_3_ 0.02 g, K_2_SO_4_ 2.2 g, MnSO_4_.7H_2_O 0.3 g, FeSO_4_ 0.1 g, MnSO_4_.5H_2_O 0.1 g, ZnSO_4_.7H_2_O 0.08 g, CuSO_4_ 0.08, fructose 1.5 g.

In the current work, the microorganisms have been purchased from the Persian collection such as *RTCCR 77 A*. *Ralstonia* eutrophic (PTCC 1615), *Rhodococcus* erythropolis (PTCC 1767), *Thiobacillus* ferrooxidans (PTCC 1646), *Thiobacillus* thiooxidans (PTCC 1717). A society of pure cultures or colonies can be made in broth medium or slants. Also, a microorganism colony has been isolated from the Iraqi heavy sour crude oil suplemeted by “9k” medium.

### Analysis and treatment methods

The important equipment used in the current study is as shown in these both sections. The main equipment for the treatment of desulfurization were the incubators as shown in table below. Test the content of sulfur before the experiment. Experimentally, types of sulfur compounds in crude oil should be determined as total content or detailed compositions by using X-ray before and after the treatment. Also, some other tests can be done as the density and then calculation of API.

For the incubation purpose, IKA^(R)^ ks 4000i, HYSK Shaking Incubator, SANYO CO_2_ Incubator compatible with Incubator Shaker. STIK oven was used for the evaporation and purification of sample to prepare it for the final test. Trans Instruments BP 3001 and 3020 were used to measure both pH and electric conductivity (EC). The sterilizer Reuhan Teb was used to sterilize the mediums before inoculation and incubation. The centrifuge Duna Velocity 14 was used in the separation of water oil phases according to ASTM 1796. TSN 6 200 sulfur elemental analyzer (NORDTECH, X-Ray (in Tehran lab) was used to test the total sulfur in the crude oil.

The experimental work was proceeded for all the experiments designed by review-based to determine the significant effect of both microorganisms (microorganisms) and culture chemical medium, or Minitab-based design by Definitive blocks to find the optimum conditions for the best microorganisms. **The** design of experiments was coupling many types of microorganisms with many probable beneficial mediums. *Thiobacillus ferrooxidans* was examined with the mediums PTCC 2, 105, 106, 132; *Rhodococcus* erythropolis was examined with MG-medium (SFM), MS, and BSM; RTCC R77 and YFC were examined with the mediums PTCC 2 and 106 respectively. Pretreated Sample (oil, water, and surfactant) with volume compatible with DoE to be enough in the final tests (2 ml) at least for testing the total sulfur. The compositions were: oil 5 ml, inoculation microorganism seed culture 5 ml, incubation chemical medium 50 ml.

#### Isolation

Isolation: Microorganisms colony was isolated from the crude oil by using Medium 106 ‘9k’ (100–150 ml) prepared as three solutions all chemicals without FeSO_4_, only FeSO_4_, agar. The source of sulfur was crude oil activated with ore concentrate. The isolated colony biomisture can be named as a yellow form colony.

#### Emulsification

The emulsifier here was Tween 80. Few amounts of surfactant can be achieved by adding some Tween with water and use droplets to pick from this diluted Tween. Tween is preferred on SDS due to no sulfur in its structure to avoid the probability of its effect on final reading or results if it does not sediment in centrifuge.

#### Acidity

It is preferred to set the pH value before sterilization to simplify procedure and keep or assure sterilized environment and sterilized pins. Maybe, this step requires partitioning of the samples to many Erlenmeyer for simplifying the step. Many adjusters are there such as HCl, H_2_SO_4_, H_3_PO_4_, and NaOH. That H_2_SO_4_ is not preferred to be used because it contains S. Usually HCl was used here.

#### Sterilization

Sterilization of chemical mediums and lab tools as the cotton and glasses can be occurred in autoclave. Medium 105 was sterilized in autoclave without FeSO_4_ avoiding sedimentation. FeSO_4_ was sterilized alone in an autoclave and then mixed with remaining mixture, or can be added by using a micro-filter (micro syringe) (4 μm). The sterilization conditions were setted as the pressure 15 psi, temperature 120 C for 20 min. In the xase of FeSO4, the temperature was setted as 100 C only for 5 to 10 min to avoid its sedimentation in the culture medium.

#### Inoculation

Inoculation for fermentation was about 2 days: Erlenmeyer contains (25–100) ml of inocula. It’s not obligatory that the medium of culture is the same as the medium of fermentation. For adaptation, as mentioned before, some drops or 0.5 ml of crude oil can be added. Time of seed culture inoculation must be the same in all experiments in order to keep constant OD for all, let it (t 2 day). Incubator setting for fermentation inoculation T 30–35 C, speed of rotation 150 rpm, and time 2 day.

#### Incubation

This step represents the main BDS reaction. That’s of medium (50 ml) and sample (5 ml) wanted to be treated and inoculated biotreater (microorganisms) (5 ml) about 4% [28].

#### Separation

The separation of treated samples (oil/water). That’s of treated sample from the previous mixture using gravity settling (decanter flask for about 20–30 min), chemical solvent or cleaner (reverse emulsifier or demulsifier such as hepate), heating (oven) (T 80 C, t 1 day) in case of gel especially in emulsions samples No. (6, 11, 22) and 20, and/or. Also, the medium after separation may contain some oil which can be separated by centrifugation actually (Sr 1Trpm, t 10 min) or (T 20 C, Sr 78269 g, t 1 h) [28] which may be after heating. Practically and usually, 500 rpm and (t 10 min) can be used mostly to separate most samples.

## Results and discussion

In this part of the current study, the results of treatment are run on a lab scale. Whereas the main step is the incubators units. The observations, readings, results, and discussion are monitored and tested during the time periods of three days in order to see the profile changes.

### Results

The results cab be noted as the isolates, desulfurization, and checking the presence or absence of BDG simultaneously with the BDS of crude oil.

#### Biopreparation by isolation of yellow form colony

**Fig 1:**
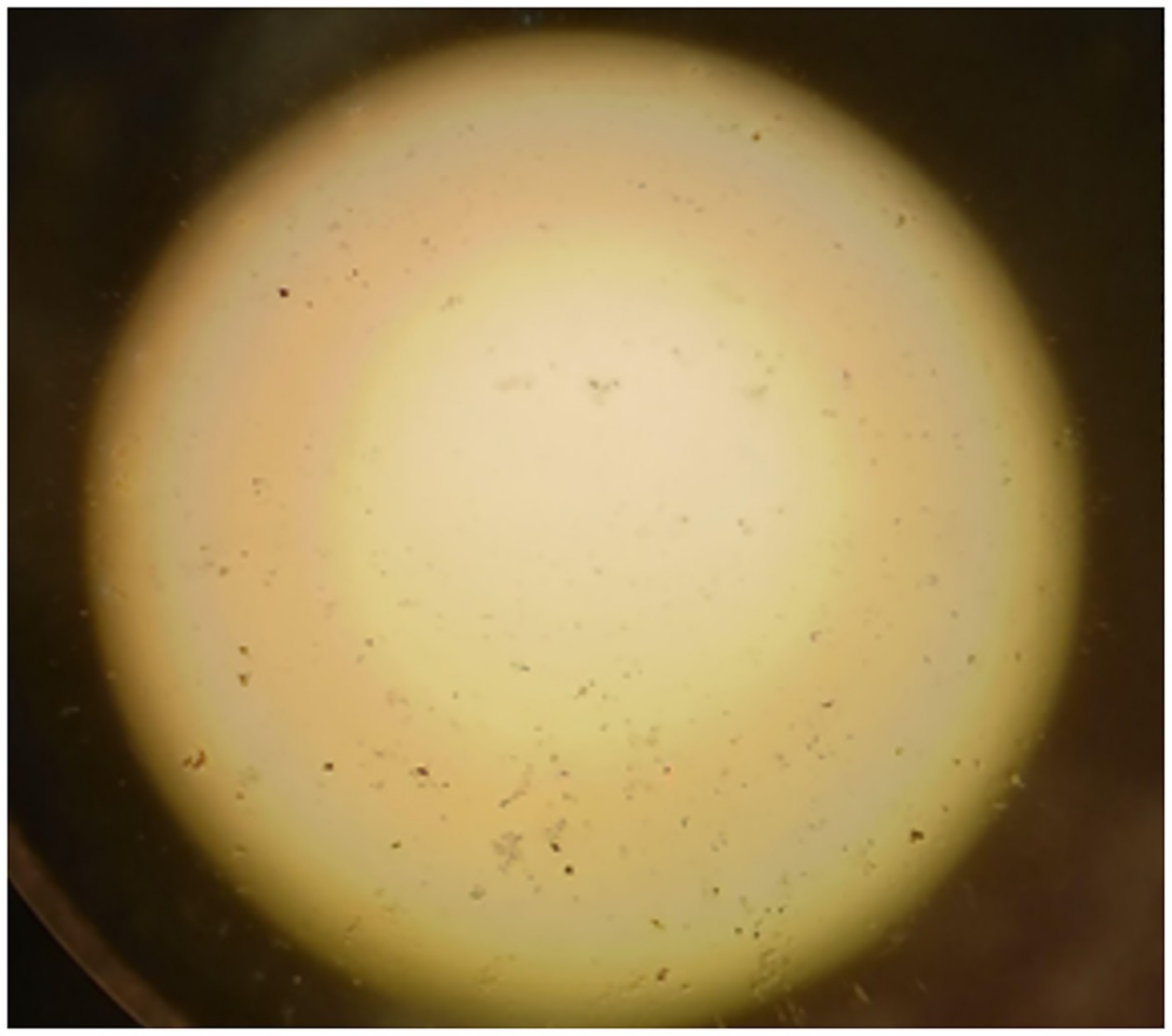
Photograph for the yellow form colony isolated from the crude oil supplemented by 9k medium. Also, in order to study the septic effect of bacillus strains as known colony, a biomixture of known bacteria (*Acidithiobacillus ferrooxidans* and *Acidithiobacillus thioxidans*) was made.

A yellow bacteria colony, as shown in (Fig 1), was isolated from the crude oil by applying PTCC 106 ‘9k’ medium supplemented by nutrient agar (1.5%) in incubation temperature 35 C for 3 day. The generated isolate was named as the yellow form colony (YFC).

#### Significance of Biodesulfurization of crude oil

**Fig 2:**
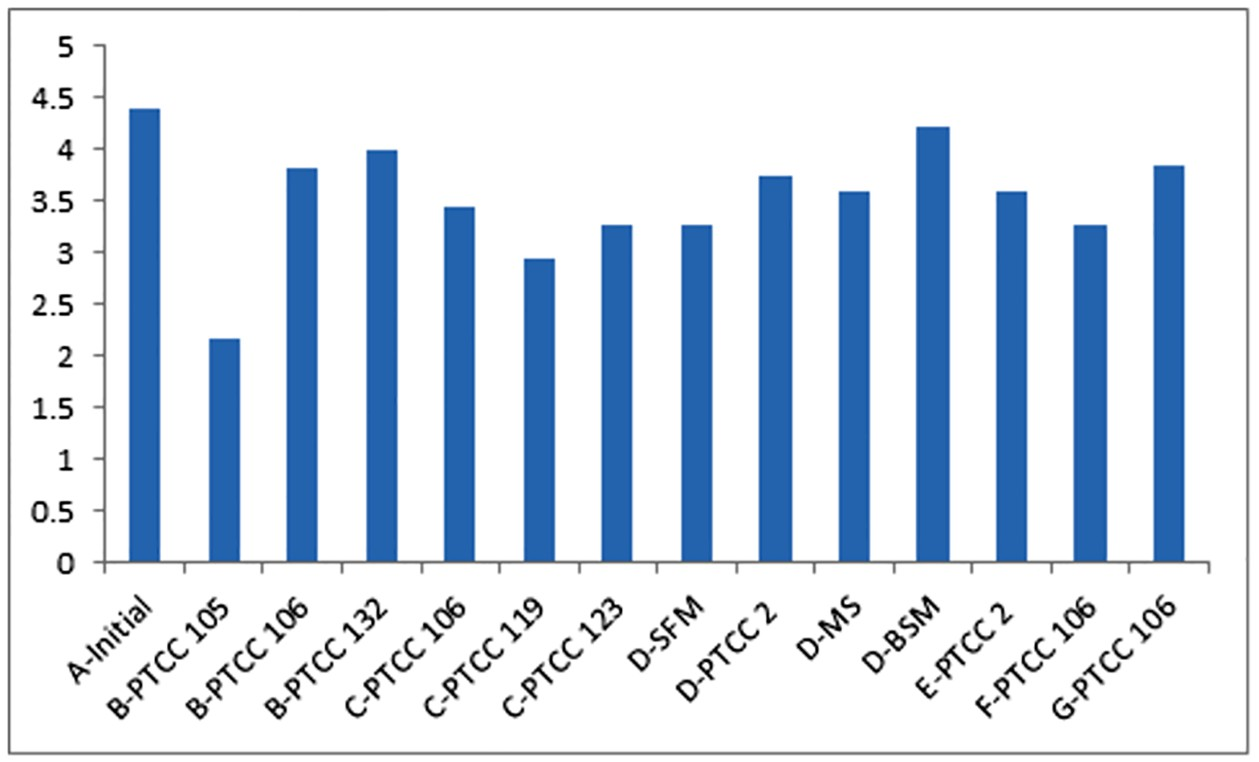
Effect of microorganism treaters and environmental mediums on the BDS of whole sour heavy crude oil. (A) Initial content, (B) *Acidothiobacillus* ferrooxidans, (C) *Acidothiobacillus* thioxidans, (D) *Rhodococcus* erythropolis, (E) *Ralstonia* eutropha RTCC 77, (F) biomixture (B+C), (G) Isolated colony (yellow form colony).

In this biotreatment, the results of 13 experiments were found. The following Table 1 shows these observations, readings, and results according to time of inoculation and incubation were 2 and 3 day respectively, as shown in Table 1 and (Fig 2).

**Table 1:** The efficiencies of both microorganisms and environmental mediums on the BDS of whole sour heavy crude oil.

Microorganism	Medium	Sources Ratio of energy/N/P/Mg	Desulfurization efficiency %	Physical Appearance
*Acidothiobacillus* ferrooxidans	PTCC 105	10/0.12/0.12/0.12	50.91	Demulsified
	PTCC 106	10/3/10/0.75	13	Homogenous emulsion
	PTCC 132	10/0.066/13.5/0.105	9	Demulsified
*Acidothiobacillus* thioxidans	PTCC 119	10/0.1/3/0.1	22	Demulsified
	PTCC 106	10/3/10/0.75	33.12	Demulsified
	PTCC 123	10/4/0.5/0.5	25.53	Demulsified
*Rhodococcus* erythropolis	SFM	10/2/12/0.4	25.57	Mostly demulsified
	MS	10/0/26.7/0	14.77	Emulsion
	BSM	10/100/100/50	18.18	Demulsified
	PTCC 2	-	4.27	Mostly demulsified
*Ralstonia* eutropha RTCC 77	PTCC 2	-	18.272	Homogeneous emulsion
Mixed colony (Th.F+Th.T)	PTCC 106	10/3/10/0.75	25.64	Demulsified
Isolated colony (YFC)	PTCC 106	10/3/10/0.75	12.61	Emulsion

Whereas, the efficiency is defined as the change in sulfur content divided by the initial sulfur content.

#### Checking Uni-objective

**Fig 3:**
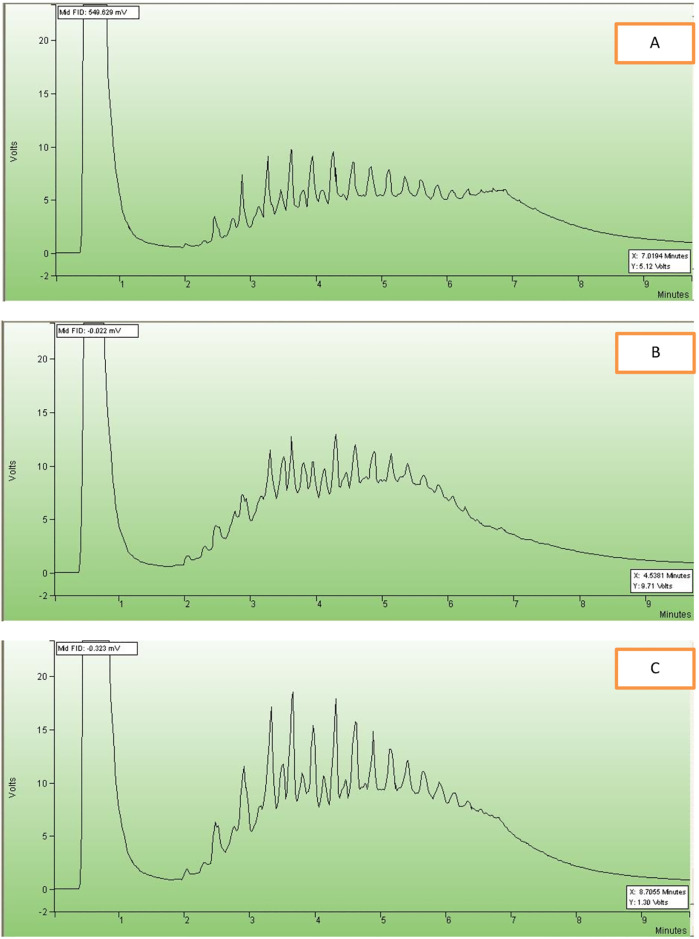
Compositions analysis for oil samples. (A) untreated oil, (B) treated crude oil by *Rhodococcus* erythropolis IGTS8, and (C) *Thiobacillus* ferroxidans.

The BDS without BDG was checked for the treated samples by using GC-FID. Whereas the similarity among the samples before and after treatment proved that BDS was not accompanied by BDG or reducing the caloric energy of hydrocarbons. Fig 3 shows that similarity.

#### Optimization of BDS operation conditions

The experiments have benn designed by Definitive method as shown in the following Table 2. The final sulfur content of crude oil have been found by measuring the total sulfur by ASTM D4294 as shown in the following Table 2.

**Table 2:** Design of experiment by Definitive method and results by both *Rhodococcus* erythropolis *IGTS8* and *Thiobacillus* ferroxidans.

n	Speed	Temperature	pH	Surfactant	OWR	*Rhodococcus* erythropolis IGTS8	*Acidithiobacillus* ferroxidans
1.	200	50	5	1	5	18.18	98.48
2.	200	50	1	0	10	52.52	22.14
3.	200	40	9	0	5	37.5	15.15
4.	200	30	9	0.5	10	4.24	2.84
5.	200	30	1	1	7.5	7.95	17.16
6.	100	50	9	0	7.5	26.58	13.64
7.	100	50	1	0.5	5	97.9	36.36
8.	100	40	1	1	10	18.18	57.66
9.	100	30	9	1	5	73.4	72.73
10.	100	30	5	0	10	22.73	47.40
11.	150	50	9	1	10	15.55	21.59
12.	150	40	5	0.5	7.5	22.73	18.83
13.	150	30	1	0	5	81.82	57.34

### Discussion and analysis

#### Explanation of Phenotypic Appearance of isolated colony

Mostly the colony growing on Fe_2_SO_4_ medium contained the microorganisms preferring sulfuric mediums such as the *Acidithiobacillus* ferrooxidans. Therefore, the color is close to the yellow because the celluar morphology is affected with that medium.

#### BDS efficiency and effect of weight ratio of sources of energy/N/P/Mg

It was found that the efficiency was related to the type of microorganism, biopurity status (septic, semiseptic, or aseptic), and the enviromental status (amounts and types of nutrients). Table 1 shows the results of significant study of microorganism effect on the desulfurization of crude oil. The most efficient microorganism and mediums were *Thiobacillus* ferroxidans with PTCC 105, *Thiobacillus* thiooxidans with PTCC 119, *known mixed colony* with PTCC 106, *Rhodoococcus* erythropolis with MG-medium (SFM). It was found that the ThF prefers a minimized ratio of E/N/P as shown in the table. The specificity of *Thiobacillus* ferroxidans toward the source of energy is the most important and has the highest effectiveness than other sources of N, P, and Mg.

It was found that the pure microorganisms can be developed by adding other microorganisms as in the case of *Thiobacillus* ferrooxidans and *Thiobacillus* thiooxidans. The biomixtiture is rather to be with regarding the preferred medium for each microorganism due to the pur *Thiobacillus* ferrooxidans with PTCC 105 achieved desulfurization more than both of the *Thiobacillus* thiooxidans and the relative colony.

While, the isolated colony is not purified, therefore, this leads to a lower efficiency of BDS, because it was applied in septic conditions.

When the microorganism *Rhodococcus* erythropolis was applied, the mediums effect ranked according to this series SFM, MS, NB, and BSM respectively. The medium free of sulfur (or medium whose difficulty in withdrawal of sulfur) with oil in treatment led to finding microorganisms working on dissociation of C-S bonds in crude oil and necessity of presence of crude oil for adaptation in seed culture, fermentation, main treatment of incubation.

The differentiation of efficiencies in desulfurization of crude oil can be significantly due to the differences in the ratio of sources of C/N/P affecting the growth and treatment of *Rhodococcus* erythropolis, i.e., the ratio among the carbohydrates/nitrates/phosphates in *Rhodococcus* strain [29], whereas, its 5/1/6 in SFM, while 1.5/0/5 in MS, and 0.1/1/1/0.5 in BSM as shown in Table 2. or generally in the various strains [30] and the cell volume [31].

Also, the efficiency varies due to the specificity of microorganisms according to the various types of HCS in the crude oil submitted to the biotreatment. Whereas *Rhodococcus* erythropolis IGTS8 has a tendency of specificity towards DBT more than alkylated forms and BT respectively, Also, each microorganism has special selectivity for which sources of sulfur [22,32,33]. Also, the various availability of sources of sulfur compounds generate a competitive in desulfurization, therefore the efficiency with multiple substrates is less than single substrate [10,26,34]. Therefore, the microorganisms desulfurize crude oil and its derivative with different efficiencies according to the presence of these HCS in fractions [32].

In general, the pure rate of BDS is low, therefore electrokinetic or sonochemical fields can be used to increase the bioreaction rate [15].

The operation conditions can be seen from the Table 2 to select the optimum conditions which give the highr desulfurization ability.

#### Checking objective unity, modeling, analysis and optimization

**Fig 4:**
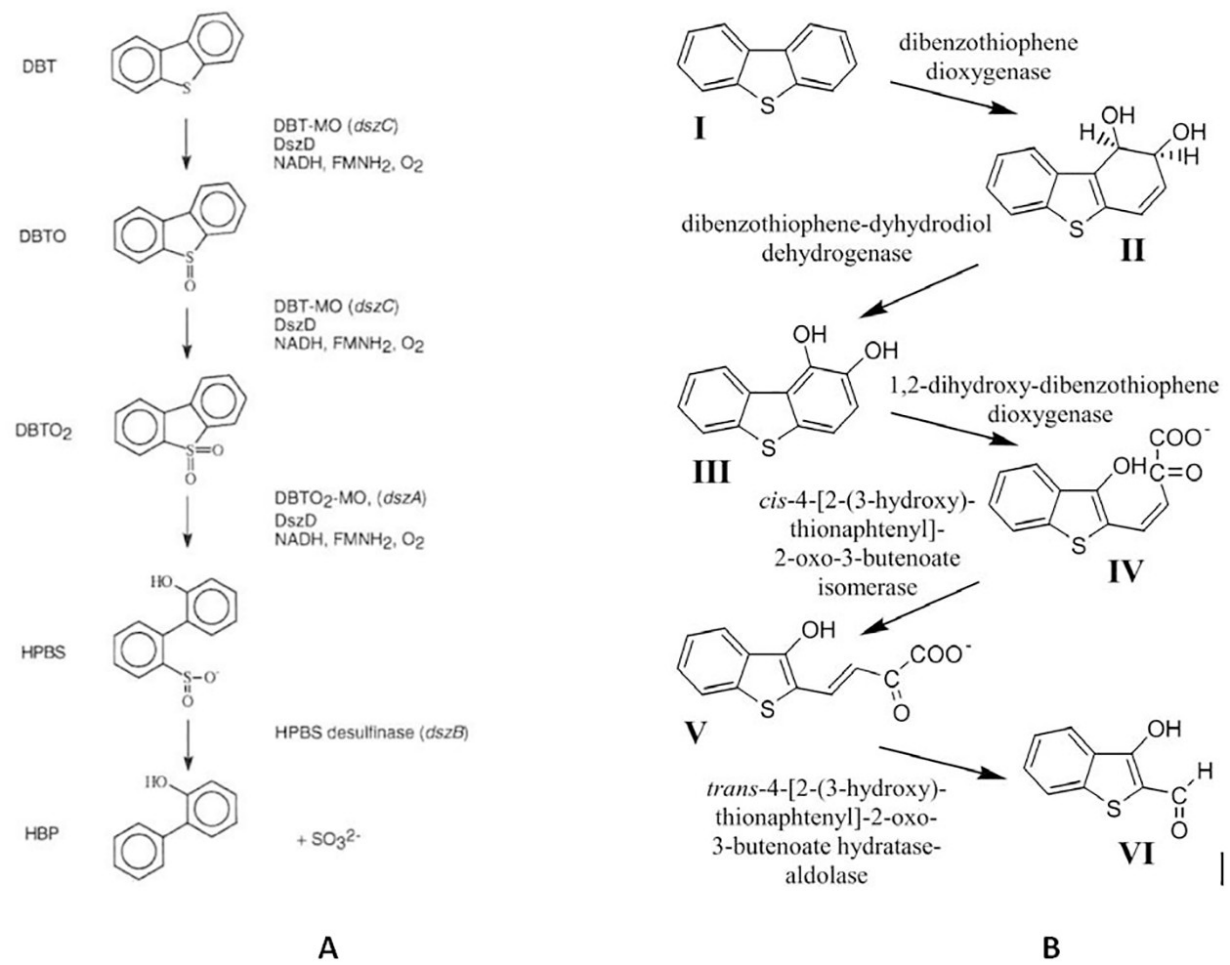
Metabolism pathways. (A) Biodesulfurization versus (B) biodegradation pathways.

This showed that those microorganisms preferred the mechanism such as the 4S pathway rather than the destructive pathways [35,36]. This leads towards the BDS kinetic model, away from the BDG models such as Kodami model. The real model of our system is multiple metabolic-based models, for simplification, a single model can be seen here [37]. The following Fig 4 shows comparative pathways between the BDS vs BDG for a model compound represented by DBT.

**Fig 5:**
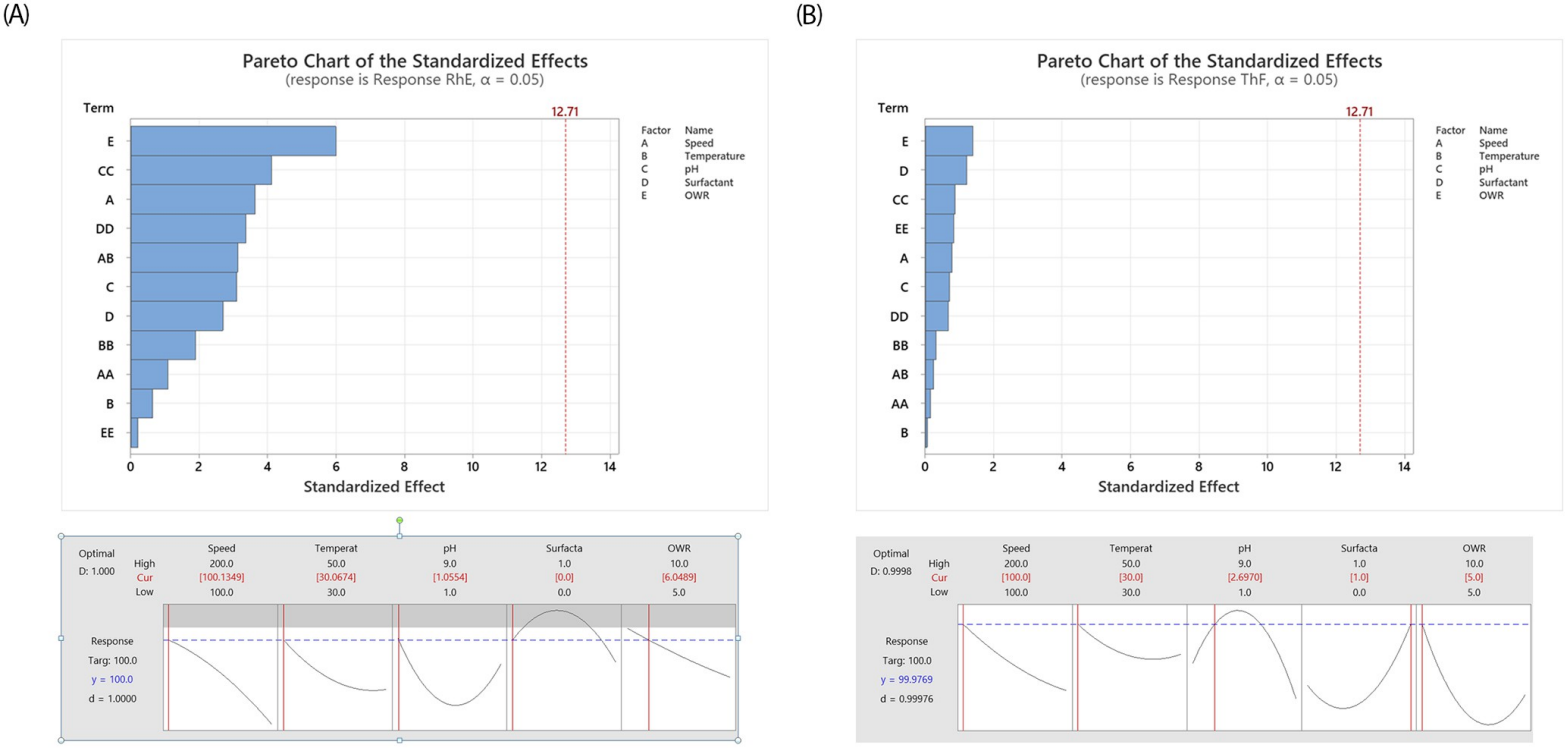
Response analysis and optimization of the operating conditions. (A) *Rhodococcus* erythropolis IGTS8 and (B) *Acidothiobacillus* ferroxidans.

The optimum conditions can be found by applying the Minitab- RSM as following Fig 5. The optimal efficiencies were 100% and 99.98% for *Rhodococcus* erythropolis IGTS8 and *Acidithiobacillus* ferrooxidans respectively. These results are the best co mparative to the previous efforts in BDS of fuels even those containing deep biological internals [17,38].

## Conclusions

The microorganisms have significant effect differences as biotreaters in the BDS treatment, whereas, the best efficiencies have been gotten by *Rhodococcus* erythropolis and *Thiobacillus* ferrooxidans in SFM and medium 105 with desulfurization efficiencies 19.74% and 47% respectively during 3 days in the primary stage. The microorganisms have no significant effect as biotreaters in the BDS treatment were RTCCR 77 A. eutrophic, and the yellow form colony (YFC) with desulfurization efficiencies less than 4% for both of them. Also, the rank of achievement of required objective was found as follows: Rhodococcus erythropolis, Thiobacillus ferrooxidans, RTCCR 77 A. eutrophic, and yellow form colony (YFC). The optimum ranges of operation conditions have been limited by using Definitive method and the optimum values can be found be using the response surface method.

## Supporting information

S1 File (DOCX)

S2 File (DOCX)
