# Supplementary material for: Optimization of Biodesulfurization of sour heavy crude oil
Source: PLoS One. 2023 Apr 4;18(4):e0283285. doi: 10.1371/journal.pone.0283285 (PMC10072378; doi:10.1371/journal.pone.0283285)
Supplement: S1 File — (DOCX) [file pone.0283285.s001.docx]

| **Table** DoE based on the liertaure review survey for studying the for significant parameters | | |
| --- | --- | --- |
| **n** | **Biomass** | **Medium** |
|  | *Acidothiobacillus* ferrooxidans | PTCC 105 |
|  |  | PTCC 106 |
|  |  | PTCC 132 |
|  | *Acidothiobacillus* thioxidans | PTCC 119 |
|  |  | PTCC 106 |
|  |  | PTCC 123 |
|  | *Rhodococcus* erythropolis | SFM |
|  |  | MS |
|  |  | BSM |
|  |  | PTCC 2 (NB) |
|  | *Ralstonia* eutropha RTCC 77 | PTCC 2 |
|  | Mixed colony (Th.F+Th.T) | PTCC 106 |
|  | Isolated colony (YFC) | PTCC 106 |

| **Table** DoE based on the Definitive Method for studying the optimum parameters | | | | | |
| --- | --- | --- | --- | --- | --- |
| **n** | **rpm** | **T** | **pH** | **OWR** | **Tween** |
|  | 150 | 50 | 9 | 10 | 1 |
|  | 150 | 40 | 5 | 7.5 | 0.5 |
|  | 150 | 30 | 1 | 5 | 0 |
|  | 200 | 50 | 5 | 10 | 0 |
|  | 200 | 50 | 1 | 5 | 1 |
|  | 200 | 30 | 9 | 7.5 | 1 |
|  | 200 | 30 | 1 | 10 | 0.5 |
|  | 200 | 40 | 9 | 5 | 0 |
|  | 100 | 50 | 9 | 5 | 0.5 |
|  | 100 | 50 | 1 | 7.5 | 0 |
|  | 100 | 30 | 9 | 10 | 0 |
|  | 100 | 30 | 5 | 5 | 1 |
|  | 100 | 40 | 1 | 10 | 1 |

| **Table** Evaluation tests of crude oil BY GC-FID ASTM 2887 | | | |
| --- | --- | --- | --- |
| **Component** | **Wt %** | **Component** | **Wt %** |
| **C1** | 0 | C18 | 2.819 |
| **C2** | 0 | **C19** | 2.9 |
| **C3** | 0.11267 | **C20** | 2.903 |
| **I-C4** | 0.6696 | **C21** | 2.859 |
| **N-C4** | 0.66316 | **C22** | 2.523 |
| **Neo-C5** | 0 | **C23** | 2.272 |
| **I-C5** | 0.39296 | **C24** | 2.219 |
| **N-C5** | 0.66316 | **C25** | 2.19 |
| **C6** | 1.582 | **C26** | 2.084 |
| **C7** | 2.156 | **C27** | 2.16 |
| **C8** | 2.45 | **C28** | 2.215 |
| **C9** | 2.913 | **C29** | 2.052 |
| **C10** | 3.141 | **C30** | 2.223 |
| **C11** | 3.061 | **C31** | 2.377 |
| **C12** | 2.936 | **C32** | 1.943 |
| **C13** | 2.88 | **C33** | 2.078 |
| **C14** | 2.77 | **C34** | 1.853 |
| **C15** | 3,267 | **C35** | 1.829 |
| **C16** | 2.997 | **C36+** | 23.824 |
| **C17** | 2.929 | **TOTAL** | 100 |

| **Table** Evaluation tests of crude oil | | | |
| --- | --- | --- | --- |
| **Test** | **Value** | **Unit** | **Remarks** |
| **Temperature** | 30 | C |  |
| **Density at T** | 0.902 | g/ml | ASTM D1298  ASTM D5002 |
| **Density at 15 C** | 0.9116 | g/ml |  |
| **API gravity** | 23.6 |  |  |
| **Salt content** | 105 | mg/l | IP 77 |
| **Water content** | 0 | Vol % | ASTM D4006 |
| **Sediment** | 0 | Vol % | ASTM D473 |
| **Emulsion** | 0.1 | Vol % | ASTM D4007 |
| **Sulfur content** | 3.86 | Wt % | ASTM 3.86 |

**
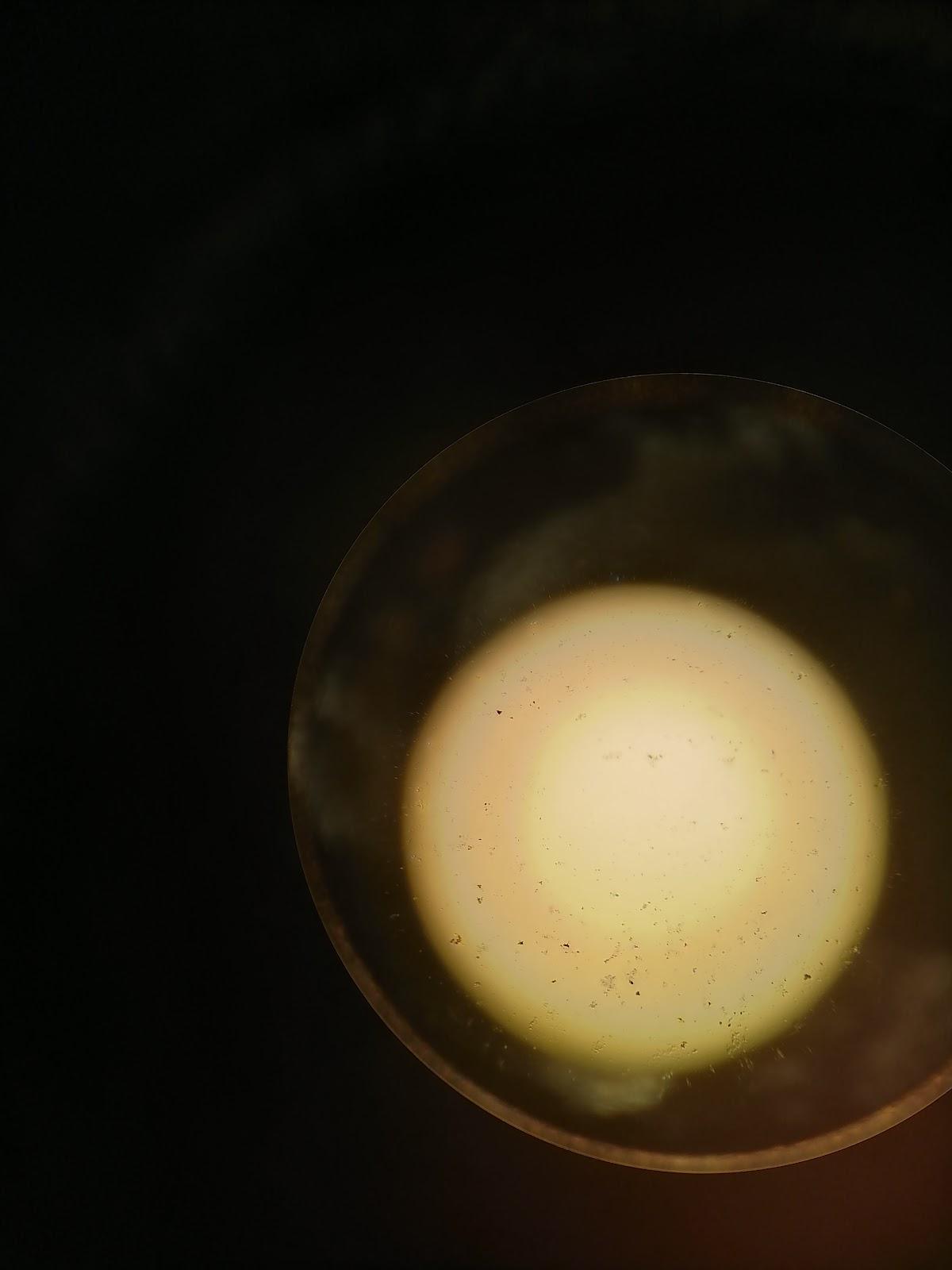
**

**Figure (4.2)** Yellow form colony isolated from crude oil

| **Table** Preparation of bio-mixtures of known and unknown colonies | | |
| --- | --- | --- |
| **Colony** | Medium | Supplier or isolation description |
| **Defined Mixed Colony of (Th.F+Th.T)** | Mixed growth cultures | Tehran University |
| **Undefined Mixed Colony** | 9k medium | Yellow form colony (YFC) was isolated by this medium from the crude oil |

| **Table** The efficiencies of both microorganisms and environmental mediums on the BDS of whole sourheavy crude oil (4.4 %) in the significance study | | | | | |
| --- | --- | --- | --- | --- | --- |
| **Microorganism** | **Medium** | **Sources Ratio of energy/N/P/Mg** | S_final_ | **Desulfurization efficiency %** | **Physical Appearance** |
| *Acidothiobacillus* ferrooxidans | PTCC 105 | 10/0.12/0.12/0.12 | **2.16** | 50.91 | Demulsified |
|  | PTCC 106 | 10/3/10/0.75 | **3.83** | 13 | Homogenous emulsion |
|  | PTCC 132 | 10/0.066/13.5/0.105 | **4.004** | 9 | Demulsified |
| *Acidothiobacillus* thioxidans | PTCC 119 | 10/0.1/3/0.1 |  | 22 | Demulsified |
|  | PTCC 106 | 10/3/10/0.75 |  | 33.12 | Demulsified |
|  | PTCC 123 | 10/4/0.5/0.5 |  | 25.53 | Demulsified |
| *Rhodococcus* erythropolis | SFM | 10/2/12/0.4 | 3.274 | 25.57 | Mostly demulsified |
|  | MS | 10/0/26.7/0 | 3.60 | 14.77 | Emulsion |
|  | BSM | 10/100/100/50 | 4.212 | 18.18 | Demulsified |
|  | PTCC 2 (NB) | - | 3.750 | 4.27 | Mostly demulsified |
| *Ralstonia* eutropha RTCC 77 | PTCC 2 | - | 3.516 | 18.272 | Homogenous emulsion |
| Mixed colony (Th.F+Th.T) | PTCC 106 | 10/3/10/0.75 |  | 25.64 | Demulsified |
| Isolated colony (YFC) | PTCC 106 | 10/3/10/0.75 | 3.845 | 12.61 | Emulsion |


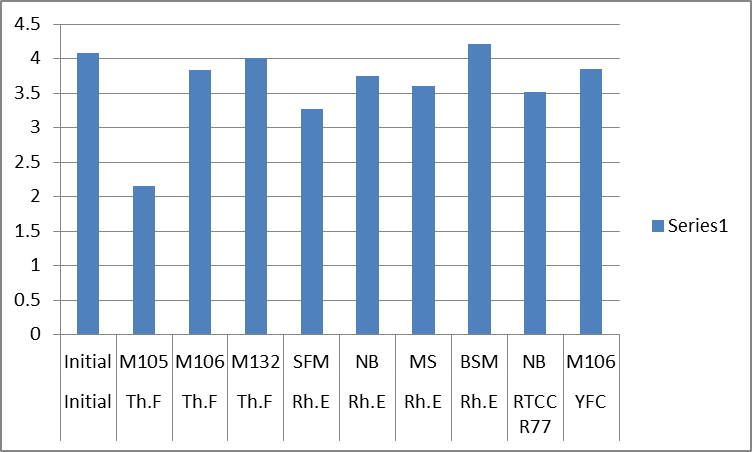


**Figure** (4.3) Results of significant study of various biomasses and media


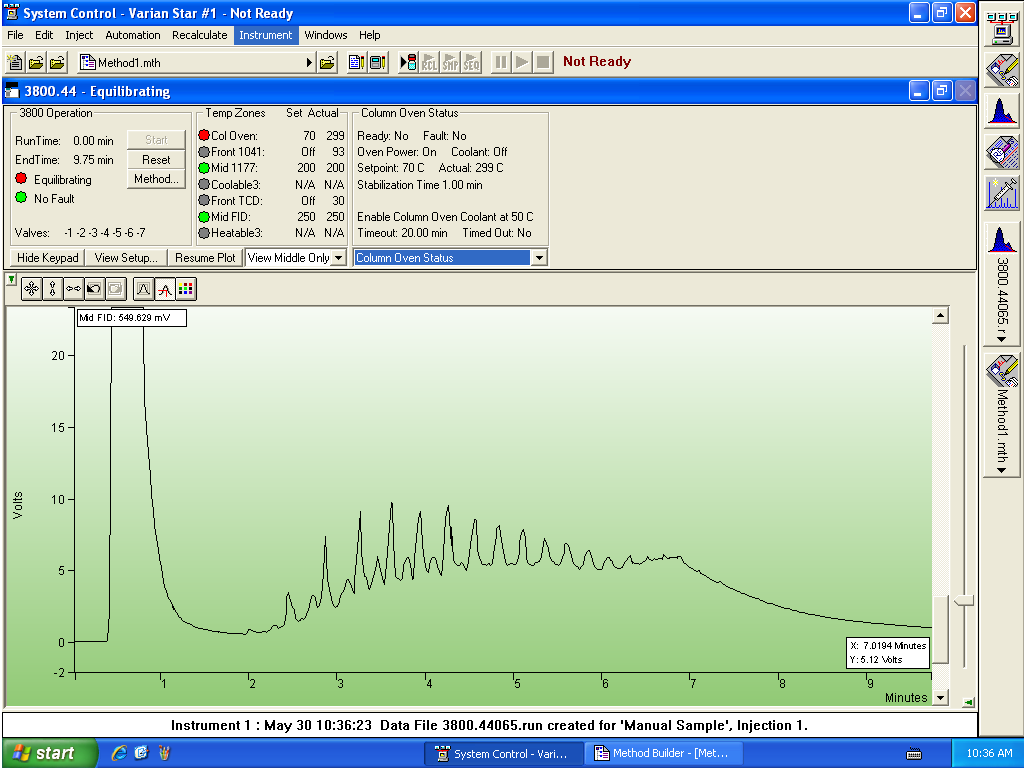


A

**
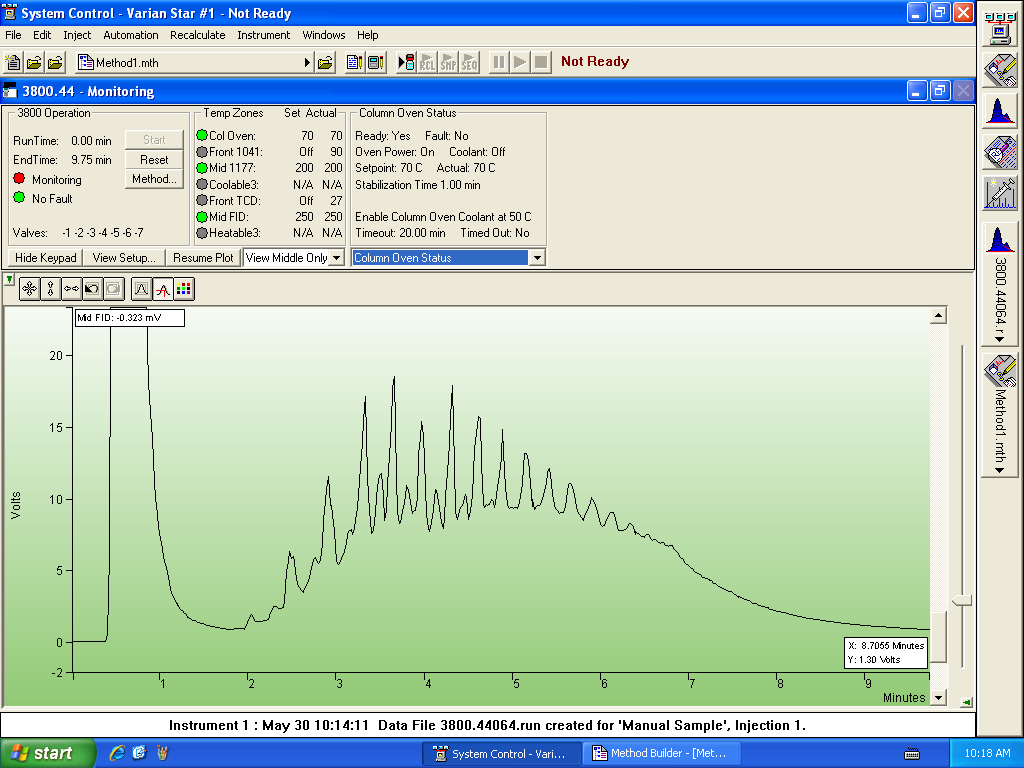
**

C

B

**
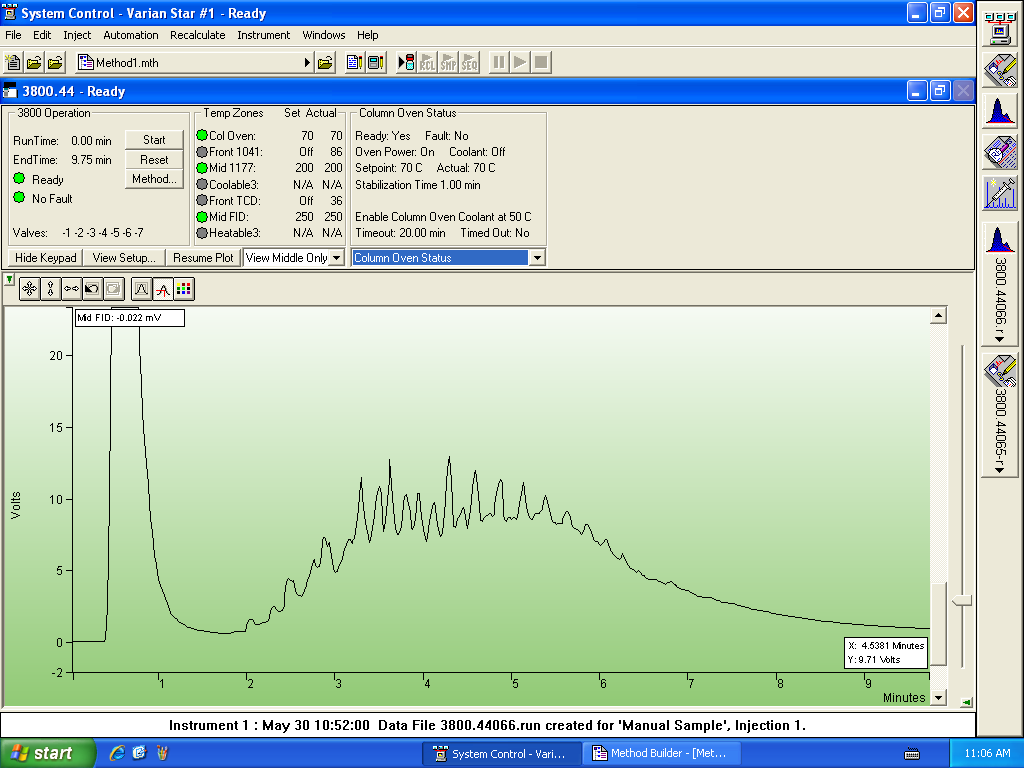
**

| **Fig** Compositions analysis of untreated (A) and treated crude oil by the significant microorganisms *Rhodococcus* erythropolis IGTS8 (B) and *Thiobacillus* ferroxidans (C).  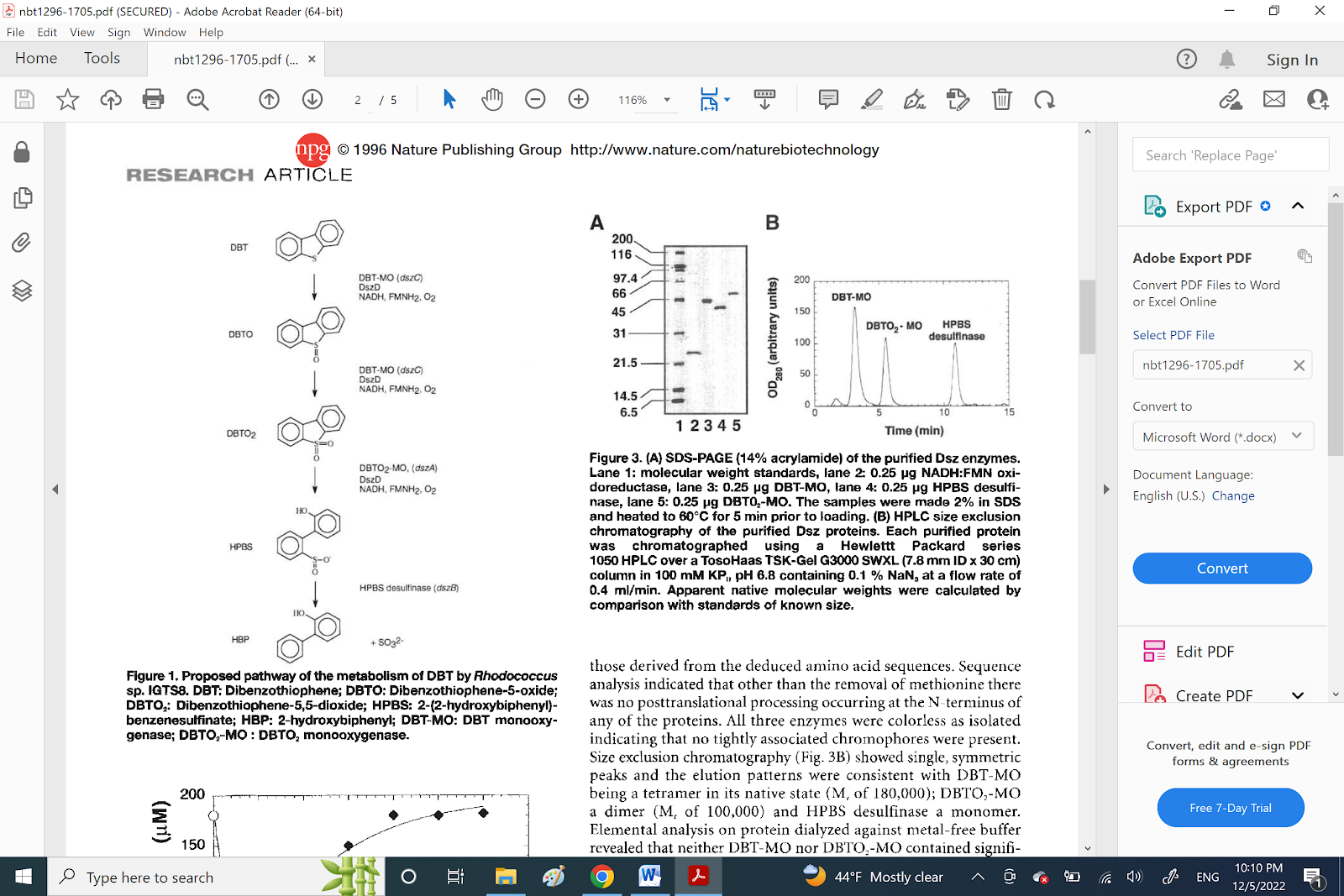 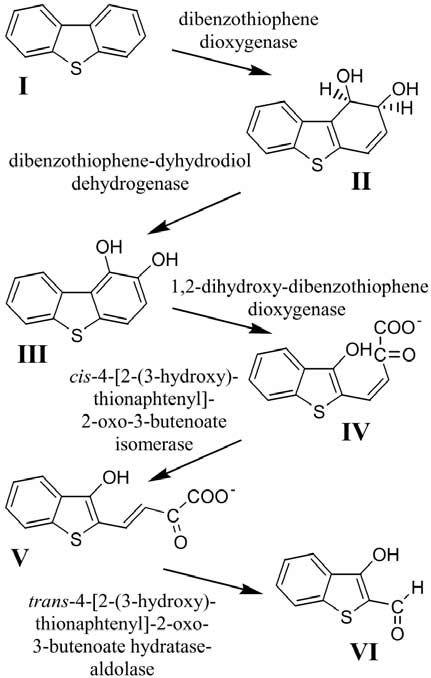  **A                                                                                           B**  **Fig** Biodesulfurization (A) versus biodegradation (B) pathways   \| **Table** Results by both *Rhodococcus* erythropolis IGTS8 and *Thiobacillus* ferroxidans \| \| \| \| \| \| \| \| \| --- \| --- \| --- \| --- \| --- \| --- \| --- \| --- \| \| **n** \| **Speed** \| **Temperature** \| **pH** \| **Surfactant** \| **OWR** \| *Rhodococcus* erythropolis IGTS8 \| *Acidithiobacillus* ferroxidans \| \|  \| 200 \| 50 \| 5 \| 1 \| 5 \| **18.18** \| **98.48** \| \|  \| 200 \| 50 \| 1 \| 0 \| 10 \| **52.52** \| **22.14** \| \|  \| 200 \| 40 \| 9 \| 0 \| 5 \| **37.5** \| **15.15** \| \|  \| 200 \| 30 \| 9 \| 0.5 \| 10 \| **4.24** \| **2.84** \| \|  \| 200 \| 30 \| 1 \| 1 \| 7.5 \| **7.95** \| **17.16** \| \|  \| 100 \| 50 \| 9 \| 0 \| 7.5 \| **26.58** \| **13.64** \| \|  \| 100 \| 50 \| 1 \| 0.5 \| 5 \| **97.9** \| **36.36** \| \|  \| 100 \| 40 \| 1 \| 1 \| 10 \| **18.18** \| **57.66** \| \|  \| 100 \| 30 \| 9 \| 1 \| 5 \| **73.4** \| **72.73** \| \|  \| 100 \| 30 \| 5 \| 0 \| 10 \| **22.73** \| **47.40** \| \|  \| 150 \| 50 \| 9 \| 1 \| 10 \| **15.55** \| **21.59** \| \|  \| 150 \| 40 \| 5 \| 0.5 \| 7.5 \| **22.73** \| **18.83** \| \|  \| 150 \| 30 \| 1 \| 0 \| 5 \| **81.82** \| **57.34** \|   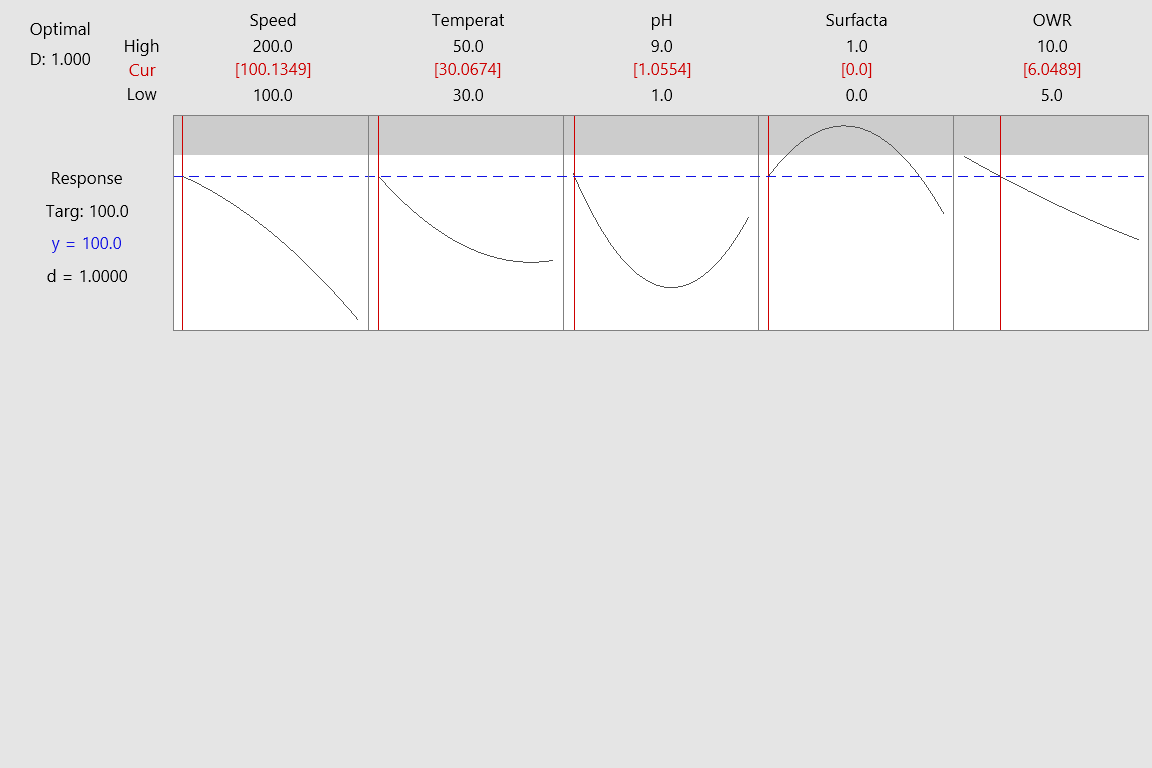  **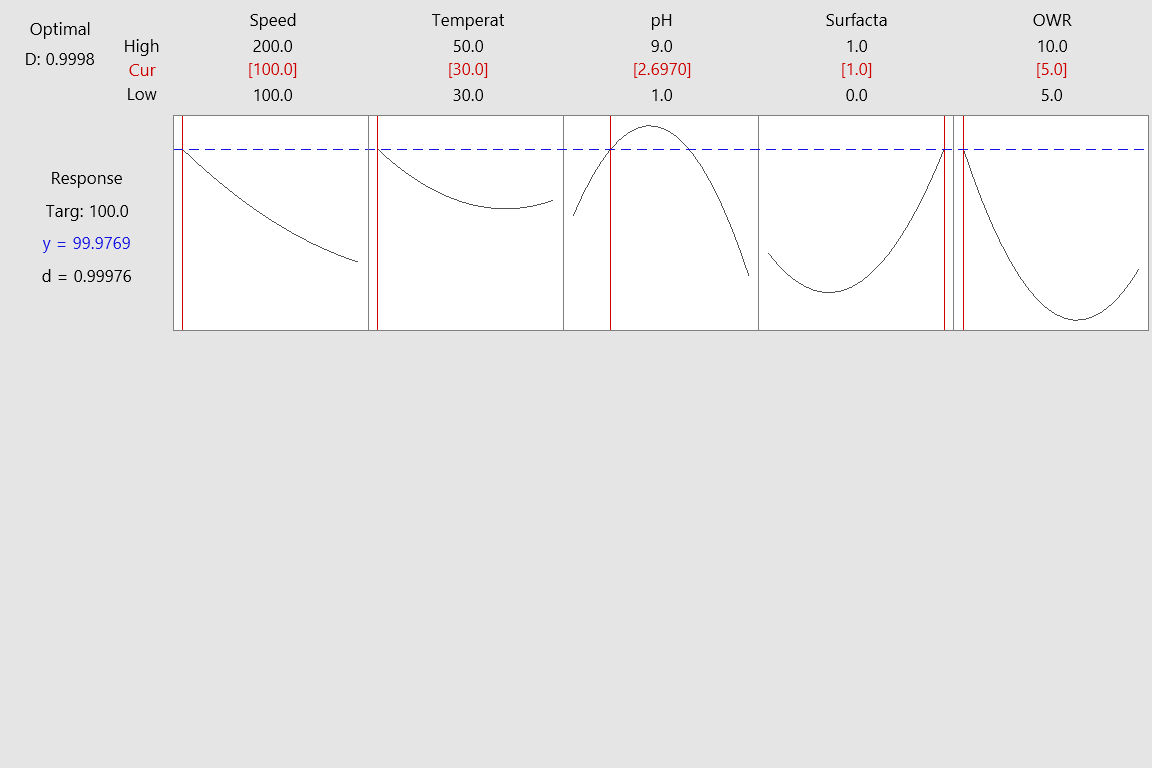**  **Fig 4.5** Response optimization for *Rhosococcus* *erythropolis* IGTS8 (A) and *Acidothiobacillus* ferroxidans (B) |
| --- | --- | --- | --- | --- | --- | --- | --- | --- | --- | --- | --- | --- | --- | --- | --- | --- | --- | --- | --- | --- | --- | --- | --- | --- | --- | --- | --- | --- | --- | --- | --- | --- | --- | --- | --- | --- | --- | --- | --- | --- | --- | --- | --- | --- | --- | --- | --- | --- | --- | --- | --- | --- | --- | --- | --- | --- | --- | --- | --- | --- | --- | --- | --- | --- | --- | --- | --- | --- | --- | --- | --- | --- | --- | --- | --- | --- | --- | --- | --- | --- | --- | --- | --- | --- | --- | --- | --- | --- | --- | --- | --- | --- | --- | --- | --- | --- | --- | --- | --- | --- | --- | --- | --- | --- | --- | --- | --- | --- | --- | --- | --- | --- | --- | --- | --- | --- | --- | --- | --- | --- |


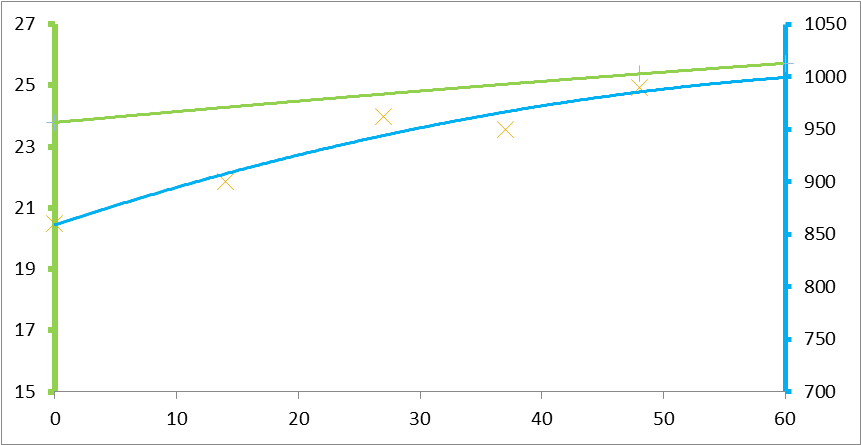

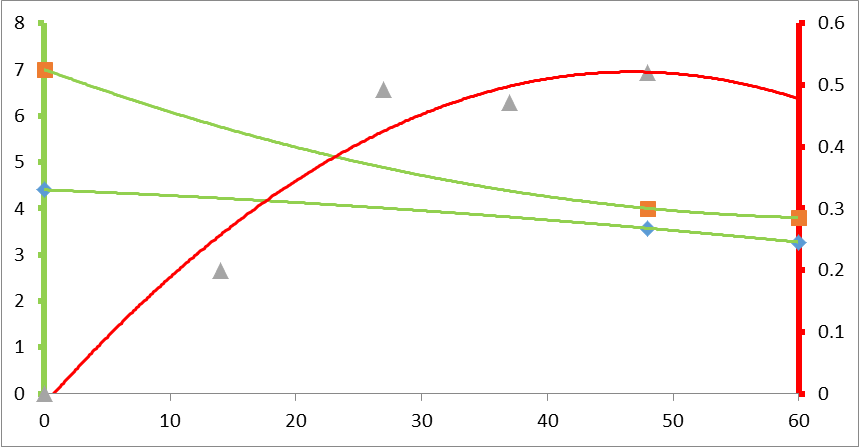

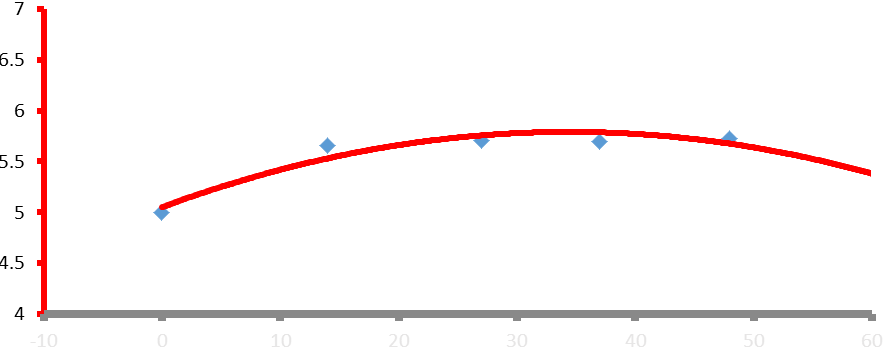


**A**

**B**


Fig The biochemical concentrations profiles [A] of reactant and product (sulfur compounds (%) [Green], salinity (mg/l*10E-3) [Blue], and microorganisms (λ) [Red]), and the chemophysical response [B] (API gravity [Green], electric conductivity [Blue], and the acidity [Red]) for the *Rhodococcus* erythropolis IGTS8 at the average operation conditions.

| **Table** Observation and results of stage I | | | | | | | | | |
| --- | --- | --- | --- | --- | --- | --- | --- | --- | --- |
| **Biomass** | **Medium** | **Day I** | **Day II** | **Day III**  **Decanter** | **S_initial_** | **S_final_** | **η_obj_** | **Av. Sp. Rate*10^3^** | **Remarks** |
| TF | 105 | Transparent totally | Two phase  Top: black oily  Bottom: aqueous 100 %.  Crude oil was more viscous than TF 132, RE SFM, RE BSM | Two phase  Top: black oily  Bottom: aqueous 100 %. | **4.079** | **2.16** | **47** | **6.269** |  |
|  | 106 | Black totally  Homogenous | Black totally  Homogenous | Black totally  Homogenous |  | **3.83** | **6.1** | **0.817** |  |
|  | 132 | Two phase  Top: black  Bottom: trans. | Two phase  Top: black oily  Bottom: aqueous 100 %. | Two phase  Top: black oily  Bottom: aqueous completely. |  | **4.004** | **1.8** | **0.245** |  |
| RE | SFM | Two phase | Two phase  Top: black oily  Bottom: aqueous 75 %. | Two phase  Top: black oily  Bottom: aqueous mostly. | 4.079 | 3.274 | 19.74 | 2.626 |  |
|  | NB | Black totally  Homogenous | Two phase  Top: black oily organic phase  Bottom: water phase without medium (Transparent) | Two phase  Top: black oily  Bottom: aqueous mostly. |  | 3.750 | 8.1 | 1.074 |  |
|  | MS | Black | Black | Black |  | 3.60 | 11.7 | 1.565 |  |
|  | BSM | Black totally  Homogenous | Two phase  Top: black oily  Bottom: aqueous mostly. | Two phase  Top: black oily organic phase  Bottom: water phase without medium (Transparent) |  | 4.212 | -3.3 | -0.434 |  |
| RTCC R77 | NB | Black totally  Homogenous | Black totally  Homogenous | Black totally  Homogenous | 4.079 | 3.516 | 13.8 | 1.839 |  |
| YFC | 106 | Black totally  Homogenous | Single phase  Homogenous  Black totally | Single phase  Homogenous  Black totally | 4.079 | 3.845 | 5.74 | 0.764 |  |

| **Table** Physical observation and separation operability after treatment (Day III) | | | | |
| --- | --- | --- | --- | --- |
| **No. Expt** | **Appearance** | **Primary separation of O/W phase**  **Dehydration** | **Secondary separation of O/Medium phase**  **Desalting** | **Remarks** |
|  |  | Decanter | Heat  PEG |  |
|  | Two phase | Decanter |  | Easy |
|  | Two phase semiblack and black | decanter | Oil gel medium was taken directly  Add hexane on it in beaker  PEG (demulsifier)  Heater or oven 77 C  Separation of HC from aqous layer  Centrifuge  (emulsion) |  |
|  |  | Decanter | Centrifuge  (emulsion) |  |
|  | Semihomogenous |  |  |  |
|  | Gel/ black  Some yellow medium |  | Centrifuge+HT (Heat) |  |
|  |  |  | Heat |  |
|  | Two phase | Decanter | Heat |  |
|  |  | Decanter | Centrifuge+HT Heat | pH=1  add NaO, heat T 90 C, centrifuge 7Trpm for t 15 min. |
|  | Single  phase | Decanter | Heat |  |
|  | Gel/ black  Some yellow medium | Decanter | Add NaOH  Heat  Centrifuge  ½ cc  Also  PEG (demulsifier)  >> separation sth  Centrifuge+HT | It was not separated by:  pH=1  add NaO, heat T 90 C, centrifuge 7Trpm for t 15 min. |
|  |  |  |  |  |
|  |  | Decanter |  |  |
|  | Decanter Two phase water and HC | Decanter | Heat |  |
|  | Two phase | Decanter |  | Easy |
|  |  | Decanter |  |  |
|  |  | Decanter |  |  |
|  |  | Decanter |  |  |
|  |  | Decanter |  |  |
|  |  | Decanter | Centrifuge+HT (Heat) |  |
|  | Two phase | Decanter | Heat |  |
|  | Gel/ black  Some yellow medium |  | Heat  Centrifuge+HT | It was not separated by:  pH=1  add NaO, heat T 90 C, centrifuge 7Trpm for t 15 min. |
|  | Two phase | Decanter | Heat |  |
|  |  | Decanter |  |  |
|  |  | Decanter | Centrifuge+HT |  |
|  | Two phase water and HC | Decanter | Heat |  |

| **Table** Indicators of separation performance in posttreatment | | | | |
| --- | --- | --- | --- | --- |
| No. Expt | Wt_empty_ g | wt_full_     g | Wt_net sample_ g | Vmedium  ml |
|  | 65.503 | 109.062 | **43.559** | 46 |
|  | 65.503 | 104.582 | **39.079** | 41 |
|  | 65.503 | 105.822 | **40.319** | 42 |
|  | 65.503 | 109.957 | **44.454** | 48 |
|  | 94.466 | 142.153 | **47.687** | 40 |
|  | 94.466 | 117.458 | **22.992** | 28 |
|  | 65.503 | 124.799 | **59.296** | 69 |
|  | 65.503 | 102.620 | **37.117** | 40 |
|  | 65.503 | 105.637 | **40.134** | 42 |
|  | 65.503 | 113.493 | **47.99** | 41 |
|  | 94.466 | 121.380 | **26.914** | 30 |
|  | 65.503 | 118.604 | **53.101** | 51 |
|  | 65.503 | 106.303 | **40.8** | 42 |
|  | 65.503 | 116.832 | **51.329** | 41 |
|  | 65.503 | 119.686 | **54.183** | 57 |
|  | 65.503 | 120.384 | **54.881** | 56 |
|  | 65.503 | 117.890 | **52.387** | 5 |
|  | 94.466 | 123.506 | **29.04** | 30 |
|  | 65.503 | 114.390 | **48.887** | 41 |
|  | 65.503 | 130.989 | **65.486** | 69 |
|  | 65.503 | 105.689 | **40.186** | 41 |
|  | 65.503 | 102.290 | **36.787** | 4 |
|  | 65.503 | 146.39 | **80.887** | 49 |
|  | 94.466 | 141.846 | **47.38** | 50 |
|  | 65.503 | 118.891 | **53.388** | 50 |
|  | 65.503 | 117.960 | **52.457** | 42.5 |

Table Monitoring the properties for the optimization experiments

| **Medium** | **TDS** | **pH** | **EC** | **EC** |
| --- | --- | --- | --- | --- |
|  | **1034** | **6.50** | **6.3** | **-** |
|  | **1751** | **0.58** | **2.85** | **16.83** |
|  | **384** | **6.25** | **40.5** | **4.38** |
|  | **740** | **1.34** | **-** | **5.83** |
|  | **392** | **2.23** | **235** | **5.29** |
|  | **347** | **5** | **212** | **4.94** |
|  | **189** | **3.40** | **176** | **3.19** |
|  | **1102** | **0.97** | **276** | **0.98** |
|  | **1251** | **1.1** | **274** | **3.91** |
|  | **381** | **4.80** | **100** | **-** |
|  | **1641** | **0.98** | **210** | **16.68** |
|  | **330** | **3.84** | **74** | **-** |
|  | **309** | **4.90** | **152** | **.88** |
|  | **1274** | **4.40** | **160** | **-** |
|  | **769** | **0.55** | **297** | **32.4** |
|  | **1345** | **3.04** | **205** | **4.40** |
|  | **1497** | **0.9** | **275** | **168** |
|  | **685** | **1.16** | **170** | **13** |
|  | **1177** | **1.87** | **225** | **13.3** |
|  | **685** | **7.25** | **125** | **-** |
|  | **1617** | **0.41** | **292** | **29** |
|  | **302** | **2.03** | **230** | **-** |
|  | **1197** | **2.52** | **195** | **23** |
|  | **730** | **0.96** | **256** | **6.87** |
|  | **1106** | **5** | **100** | **-** |
|  | **1222** | **2.20** | **237** | **14.75** |
