# Supplementary material for: Optimization of Biodesulfurization of sour heavy crude oil
Source: PLoS One. 2023 Apr 4;18(4):e0283285. doi: 10.1371/journal.pone.0283285 (PMC10072378; doi:10.1371/journal.pone.0283285)
Supplement: S2 File — (DOCX) [file pone.0283285.s002.docx]

| **Table** The efficiencies of both microorganisms and environmental mediums on the BDS of whole sourheavy crude oil (4.4 %) in the significance study | | | | | |
| --- | --- | --- | --- | --- | --- |
| **Microorganism** | **Medium** | **Sources Ratio of energy/N/P/Mg** | S_final_ | **Desulfurization efficiency %** | **Physical Appearance** |
| *Acidothiobacillus* ferrooxidans | PTCC 105 | 10/0.12/0.12/0.12 | **2.16** | 50.91 | Demulsified |
|  | PTCC 106 | 10/3/10/0.75 | **3.83** | 13 | Homogenous emulsion |
|  | PTCC 132 | 10/0.066/13.5/0.105 | **4.004** | 9 | Demulsified |
| *Acidothiobacillus* thioxidans | PTCC 119 | 10/0.1/3/0.1 |  | 22 | Demulsified |
|  | PTCC 106 | 10/3/10/0.75 |  | 33.12 | Demulsified |
|  | PTCC 123 | 10/4/0.5/0.5 |  | 25.53 | Demulsified |
| *Rhodococcus* erythropolis | SFM | 10/2/12/0.4 | 3.274 | 25.57 | Mostly demulsified |
|  | MS | 10/0/26.7/0 | 3.60 | 14.77 | Emulsion |
|  | BSM | 10/100/100/50 | 4.212 | 18.18 | Demulsified |
|  | PTCC 2 (NB) | - | 3.750 | 4.27 | Mostly demulsified |
| *Ralstonia* eutropha RTCC 77 | PTCC 2 | - | 3.516 | 18.272 | Homogenous emulsion |
| Mixed colony (Th.F+Th.T) | PTCC 106 | 10/3/10/0.75 |  | 25.64 | Demulsified |
| Isolated colony (YFC) | PTCC 106 | 10/3/10/0.75 | 3.845 | 12.61 | Emulsion |

| **Table** Results by both *Rhodococcus* erythropolis IGTS8 and *Thiobacillus* ferroxidans | | | | | | | |
| --- | --- | --- | --- | --- | --- | --- | --- |
| **n** | **Speed** | **Temperature** | **pH** | **Surfactant** | **OWR** | *Rhodococcus* erythropolis IGTS8 | *Acidithiobacillus* ferroxidans |
|  | 200 | 50 | 5 | 1 | 5 | **18.18** | **98.48** |
|  | 200 | 50 | 1 | 0 | 10 | **52.52** | **22.14** |
|  | 200 | 40 | 9 | 0 | 5 | **37.5** | **15.15** |
|  | 200 | 30 | 9 | 0.5 | 10 | **4.24** | **2.84** |
|  | 200 | 30 | 1 | 1 | 7.5 | **7.95** | **17.16** |
|  | 100 | 50 | 9 | 0 | 7.5 | **26.58** | **13.64** |
|  | 100 | 50 | 1 | 0.5 | 5 | **97.9** | **36.36** |
|  | 100 | 40 | 1 | 1 | 10 | **18.18** | **57.66** |
|  | 100 | 30 | 9 | 1 | 5 | **73.4** | **72.73** |
|  | 100 | 30 | 5 | 0 | 10 | **22.73** | **47.40** |
|  | 150 | 50 | 9 | 1 | 10 | **15.55** | **21.59** |
|  | 150 | 40 | 5 | 0.5 | 7.5 | **22.73** | **18.83** |
|  | 150 | 30 | 1 | 0 | 5 | **81.82** | **57.34** |
